# The Wife’s Domain

**Published:** 1861-07

**Authors:** 


ART. VII.—*The Wife's Domain.* By PHILOTHALOS. 12mo., pp. 162. London: John Churchill, 1860.

THE contents of this volume, as the author tells us, consist of portions of a series of discourses which have been from time to time delivered at a small establishment devoted to the relief of diseases of mothers and children, to the poor women who with their children came there for treatment. The work does not, therefore, profess to be a profound treatise on the subjects which it discusses, but rather a popular medium of information to those to whom medical technicalities would be unintelligible. Who it may be, thus disguising his identity under the name of Philothalos, it is impossible, of course, to say; but we can readily imagine how those to whom the work is especially adapted will puzzle over the pedantic name of the author.

The book is divided into four chapters, which include the duties of the Young Couple, the Mother, and the Nurse, and the Care of the Nursling. Although especially addressed to the poor and those who have to toil through an anxious life of dependence, many of the suggestions for the comfort and happiness of the married state are applicable to all conditions and all ranks of life. Yet the publisher, in issuing so neat a little work, has almost removed it from popular purchase by those of slender means, the very class the author seemed desirous of instructing.

The conditions and qualifications to be mutually exacted and established before a marriage contract is agreed upon will carry but little force, we fear, with those who are bent upon a conjugal union. They are, of course, such conditions as would seem to a writer on the subject to offer the best prospects of happiness; but what number of poor couples will be prevented by any advice writers or lecturers may give them from entering the married state by *condition second*, for example, which states that "a man ought not to marry unless he is able, either by his earnings or by independent means, to support a wife and separate establishment, which should be furnished beforehand"? In many dwellings of the poorer classes, long after matrimony has been entered into, children appear to be the main articles of furniture visible. To be sure, under such circumstances of poverty, "it is the progeny of such unions which make up the vast majority of our reprobate juvenile population. They are vagabonds from their cradle."

Moral obligations and moral delinquencies are next discussed; but the medical portions of the book are those to which we must particularly refer. The various symptoms of pregnancy are dwelt upon, the hygienic and medical points connected with that condition, the various therapeutic agents which may be safely trusted for domestic purposes in the hands of the ignorant, the subjects of diet and exercise, delivery, accidents consequent upon parturition, etc. It is all very well, perhaps, for women to know all these points thoroughly, and yet we are forcibly struck with the fact that most probably the efforts of "Philothalos" will be successful in converting a good many old women nurses into infallible practitioners of midwifery, in their own conceit, and prevent the family *accoucheur* from being sent for at times when his services would be of vital consequence. It is well enough to let women understand the causes and consequences of their complaints, but to give to the ignorant classes who seek advice at the dispensaries and hospitals formulæ for treating their own maladies is, to say the least, highly reprehensible. We are not sorry that the author has placed it in its present shape beyond the reach of those in whose hands it might be calculated sometimes to do harm.

"The best nurse for an infant," says the writer, "is its own mother." Advice on this subject to the poor, who have not the means of carrying out the physician's instructions, is "like succoring the penniless by simply telling them to get themselves clothed and fed;" for a regimen, properly selected, and suitable to the child's temperament and digestive powers, with every requisite accessory, cannot be attained by the poorer orders of the working classes, should it be necessary to appeal from the mother to artificial sources for the supply of nutriment. The little details of treatment of the new-born, as to the proper modes of washing, dressing, etc., seem almost too trifling for a medical man to discuss so minutely, although they may be of great consequence to the health of the child.

If "Philothalos" be not a medical man, his knowledge of the minutiæ of little babies' toilet must, nevertheless, commend him to the women and children whom he addresses, as a very useful person to consult upon the shape and length and quality of petticoats and sleeves and bandages. No nurse ever wrapped up a baby's legs and arms so tenderly; one would think that "Philothalos's" wife must have contributed to a large portion of the work the benefits of her own experience or that of her female friends. Nevertheless, with all these minutiæ of details, most of the advice is sound, and practically useful, unless it may induce many a poor family now and then to endeavor, even in extreme cases of peril, to dispense with the application to a competent practitioner.

